# Supplementary material for: Modelling Skylarks (Alauda arvensis) to Predict Impacts of Changes in Land Management and Policy: Development and Testing of an Agent-Based Model
Source: PLoS One. 2013 Jun 6;8(6):e65803. doi: 10.1371/journal.pone.0065803 (PMC3675089; doi:10.1371/journal.pone.0065803)
Supplement: Supporting Information S4 — The skylark ODdox as a zipped archive. (ZIP) [file pone.0065803.s004.zip › Skylark_ODdox/page2.html]

ALMaSS Skylark ODdox: Farm Management


ALMaSS Skylark ODdox
 2.0


- Main Page
- Related Pages
- Classes
- Files

Farm Management

Class for farm management

Farm class documentation following ODDox protocol

## Overview

## 1. Purpose

The Farm class is responsible for generating and managing all farm operations on a set of fields managed by a single farm.

## 2. State variables and scales

  
  
The farm unit's scale is determined by the size and location of the fields that are managed by this farm. There can be as few as one field and as many as all the fields in the landscape under consideration at the time. This is defined by the user on input and allows very realistic to very simple scenarios to be constructed.  
  
State variables:  
Farm::m\_queue - is the list of events to carry out.  
Farm::m\_fields - is a vector of fields belonging to the farm.  
Farm::m\_rotation - is the order of vegetation types that makes up the rotation.  
Farm::m\_farmtype - denotes the farmtype, e.g. 0 = ConventionalCattle  
Farm::m\_stockfarmer - whether we are stock or arable farmer (true/false respectively).  
Farm::m\_farm\_num - a farm reference number.  
Farm::m\_rotation\_sync\_index - a parameter used to potentially synchronise farm rotations between and/or within a farm.

## 3. Process Overview and Scheduling

  
The farm managers main function is to control the farm operations occuring on the fields it manages. The precise operations that occur are determined by the Crop management plans e.g. WinterWheat. The farm manager checks with each crop management plan for each field it manages and responds to requests for farm operations, returning the result as success or failure. If a field's crop management plan signals that it has terminated, then the farm manager selects the next crop in its rotation list and initiates that crop management on the field. Fields are managed for farm operations once per day, and no responses can be made by any other objects in the system between start and finish of the daily management. Hence as far as animal models are concerned the farms management is instantaneous in daily steps.


---

## Design

## 4. Design Concepts

The Farm class is not an IBM as such, but is an individual model object. As such it has its own rules for behaviour which generates emergent properties at a higher hierarchical level.  
  
**Emergence** - There are a number of emergent properties resulting from the farm managers operations. The primary one is the pattern of crops in fields across the landscape and the changes in that with time. In addtion the changes in vegetation structure and biomass, and insect biomass are a function of the precise managements carried out in each field, together with weather data. Almost all operations carried out on a field will have an impact e.g. sowing, harvest, pesticide treatments.  
**Adaptation** - The farm manager has no goals, and therefore no adaptive strategy.  
**Fitness** - The farm manager has no measure of fitness. In the future it might be possible to link success of its strategy to yield and therefore gross margin, which would provide a measure of fitness.  
**Prediction** - No prediction is carried out.  
**Sensing** - The farm manager needs to be aware of climate data to be able to make sensible crop managements e.g. no spraying pesticides in high winds.  
**Stochasticity** - Stochasticity is built in if the default setting of field by field random rotation start points is set. The precise path of the management plans will also be affected by the probability of carrying out certain farm operations, and this is also a major feature of the crop management plans.  
**Collectives** - There are no collectives as such, but the farm can be viewed as a collection of fields.  
**Observation** - The events carried out on each field can be traced and dumped to output files for checking.  
  

## 5. Initialisation

Initialisation is performed by the Landscape class (currently undocumented), by determining whether we have synchronised or random field by field start positions in the rotation. If not synchronised then each field starts at a random location in the rotation list. All farms undergo a one year dummy run in period to ensure that crops are started at the correct times of year. After this run in period the animal models will be initialised.   

## 6. Inputs

Weather and soil map inputs are required together with a list of field polygons managed by the farm.  

## 7. Interconnections

There are no submodels for the farm, but this class is strongly connected with the Crop class, and via this to all the specific management plants e.g. WinterWheat. The Crop class interacts with the Farm class via the event queue which determines what farm operations will be called. The farm operations e.g. see Farm::AutumnPlough result in changes in vegetation structure, insect biomass, pesticide and fertilizer inputs and a signal allowing other objects to determine what events have occured and react to them e.g partridge nests may be destroyed by vegetation cutting operations.


- Generated on Thu Jan 10 2013 13:15:35 for ALMaSS Skylark ODdox by
   1.8.1.1
